# Mass cytometry reveals cellular correlates of immune response heterogeneity to SARS-CoV-2 vaccination in the elderly

**DOI:** 10.1038/s41541-024-01028-2

**Published:** 2024-11-29

**Authors:** Ratnadeep Mukherjee, Linn Margrethe Eggesbø, Asia-Sophia Wolf, Ingrid Fadum Kjønstad, Guri Solum, Anthony Ravussin, Sabin Bhandari, Anna Hayman Robertson, Per Magnus, Lill Trogstad, Anja Bråthen Kristoffersen, Unni Cecilie Nygaard, Siri Mjaaland

**Affiliations:** 1 Division of Infection Control, Section for Immunology, Norwegian Institute of Public Health, Oslo, Norway; 2 Division of Infection Control, Section for Vaccine Epidemiology and Population Studies, Norwegian Institute of Public Health, Oslo, Norway; 3 Centre for Fertility and Health, Norwegian Institute of Public Health, Oslo, Norway; 4 Division of Infection Control, Section for Modelling and Bioinformatics, Norwegian Institute of Public Health, Oslo, Norway

**Keywords:** Vaccines, Viral infection

## Abstract

Heterogeneity in vaccine response, particularly in vulnerable populations like the elderly, represents a significant public health challenge. We conducted an in-depth examination of immune cell profiles before and after SARS-CoV-2 vaccination utilizing mass cytometry in a cohort of healthy Norwegian seniors (65–80 years). We have demonstrated that higher pre-vaccination frequencies of CD27^+^IgD^-^ class-switched memory B cells and subsets of CD27^-^CD24^+^CD38^+^ transitional B cells were associated with a robust vaccine response. Post-vaccination, high responders exhibited increased frequencies of IFN-γ^+^CD4^+^ T cells with antigen recall and a concurrent decrease in CCR6(+) T_H_ cell subset frequencies compared to low responders. The presence of a γδ T cell subset displaying polyfunctional cytokine responses was also associated with better vaccine response in the elderly. This in-depth profiling sheds light on inherent differences in immune cell frequencies and functions that may offer insights for targeted vaccination strategies in older populations.

## Introduction

The recent COVID-19 pandemic has placed a significant burden on global healthcare systems, with unprecedented high levels of morbidity and mortality^1^. With the large-scale deployment of multiple rounds of vaccines, many of the severe clinical manifestations of COVID-19 have been mitigated in affected individuals^2,3^. However, heterogeneity in vaccine response remains an area of concern, particularly in vulnerable populations including immunocompromised individuals and the elderly^4^.

Due to better access to advanced healthcare, there has been a rapid increase in global life expectancy. This has led to a consequent increase in the elderly population, defined as adults over 65 years of age, which currently stands at 8.5% of the world’s population and is estimated to reach 17% by 2050^5^. A decline in immune function with age, a process called immune senescence^6^, as well as increased prevalence of comorbidities and the use of immunosuppressants, makes this population specifically vulnerable to infections and severe disease. Indeed, the COVID-19 pandemic was found to affect the older population most seriously^7^ and individuals over 65 years of age had a high infection fatality rate (4.5%)^8^. Aging has also been reported to be associated with reduced response to vaccines^9^. So far, studies monitoring SARS-CoV-2 vaccine responses in the elderly have mostly utilized traditional serological analysis and targeted cellular activation assays^10–15^. Moreover, these studies have predominantly focused on older adults with associated comorbidities. Notably there has been a lack of information on inter-individual variation in immune responses to SARS-CoV-2 vaccines in healthy adults over 65 years of age. Recent work from our group addressed part of this knowledge gap by combining immune response measurements with epidemiological data in a population based Norwegian cohort of healthy seniors. We demonstrated the generation of heterogeneous, although overall robust, humoral and cellular responses in vaccinated seniors and identified epidemiological correlates of a reduced vaccine response^16^. However, given the multifaceted nature of any immune response, a more detailed exploration of various cell types and function is needed for a better understanding of the underlying mechanisms of heterogeneity in vaccine responses in the elderly.

Cytometry by time-of-flight (CyTOF) is a tool for broad and deep profiling of immune cell composition and function, applied across many clinical settings including infections and vaccinations^17–21^. Moreover, such ‘high dimensional’ approaches have revealed inter-individual heterogeneity in immune system composition as important predictors of systemic responses to disease^22^. However, a similar comprehensive analysis of cellular immunity to SARS-CoV-2 vaccination in the elderly is still lacking.

In the present study, we performed a 40-parameter deep profiling of immune cell phenotype and function to identify cellular correlates of vaccine responsiveness in selected low and high vaccine responders from a longitudinal population-based cohort of Norwegian older adults^16^. Our exploratory analysis aimed to identify both inherent differences in immune cell frequencies and function of various subpopulations before vaccination, as well as qualitative and quantitative differences after two doses of mRNA COVID-19 vaccine. Unsupervised clustering and subsequent statistical analysis revealed differences in immune cell frequencies and function between low and high responders during pre- and post-vaccination. Our results are anticipated to serve as a valuable resource for future development of targeted vaccination regimes.

## Results

### Study design

**Fig. 1:**
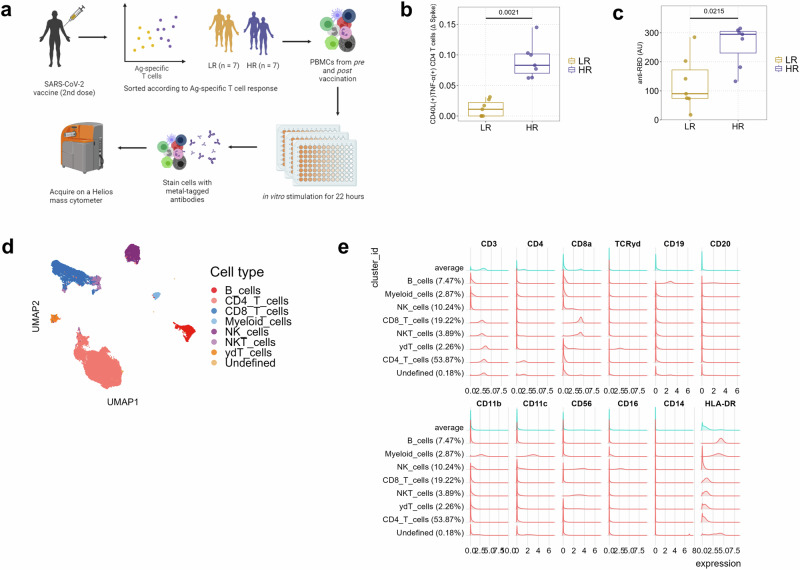
Study design and visualization of cell populations. **a** PBMCs isolated from low (LR) and high (HR) responding donors to SARS-CoV2 vaccination, before and after 2 vaccine doses, were left unstimulated or stimulated with either spike-specific peptides or Cytostim for 22 h, followed by staining with metal-tagged antibodies and subsequent acquisition on a mass cytometer. Created with Biorender (https://www.biorender.com/). **b** Spike-specific CD40L^+^ TNF-α^+^ CD4^+^ T cell frequencies in vaccine low (LR) and high (HR) responders. p values from Wilcoxon rank sum test comparing the two groups are shown. **c** Circulating levels of anti-RBD antibodies in vaccine low (LR) and high (HR) responders. Statistical significance was calculated by the Wilcoxon rank sum test. A *p* < 0.05 was considered significant. **d** Visualization of major cell clusters in a UMAP space. The clusters were manually annotated into known cell types by visual inspection of canonical marker intensities presented in the marker histogram (**e**).

We selected samples from a longitudinal population-based cohort of Norwegian healthy seniors before and after two doses of vaccine against SARS-CoV-2 (min. 14 days post-vaccination) and with no record of SARS-CoV-2 infection (Table 1)^16^. 4 out of 7 individuals in both the response groups had associated comorbidities (Table 1), with asthma and lung disease occurring together, as well as myocardial infarction and heart disease. No individual had more than two comorbidities presenting together. Response to vaccination was quantified by measuring serum levels of anti-RBD IgG, as well as by the frequencies of responding spike-specific CD40L^+^TNF-α^+^ CD4 T cells in peripheral blood upon in vitro activation by SARS-CoV-2 spike peptides, as reported previously^16^ (Fig. 1a). Based on the change in frequency of spike-specific CD4^+^ T cells after the second vaccine dose compared to baseline, we selected 14 individuals and categorized them as *low* and *high responders* according to specific T cell responses (frequency of CD40L^+^TNF-α^+^ CD4^+^ T cells lower than or greater than 0.05% (pre-vaccination – post dose 2, respectively, *n* = 7 each) (Fig. 1b). Despite displaying heterogeneity, the low responder (LR) group was found to have significantly lower amounts of circulating anti-RBD IgG than the high responders (HR) (Fig. 1c).

**Table 1:** Study sample details

Parameter	Low Responders (LR)	High Responders (HR)
N (male/female)	7 (5/2)	7 (4/3)
Median age (range), yrs	72 (66–78)	70 (69–73)
Time of sampling after vaccination in days, median (range)	33 (18–90)	27 (19–41)
Comorbidities (no./total)		
Asthma	1/7	0/7
Chronic lung disease	1/7	1/7
Diabetes	0/7	1/7
Myocardial Infarction	1/7	1/7
Heart condition	0/7	1/7
Stroke (anytime)	1/7	0/7
Chronic liver disease	0/7	0/7
Chronic kidney disease	0/7	0/7
Neurological disease	0/7	0/7
Cancer (previous and active)	0/7	2/7
Immunosuppression^a^	2/7	0/7

^a^Based on questionnaire, any one or more of: (i) Organ transplant recipient, (ii) Having an immunodeficiency, and (iii) Using immunosuppressive medications.

### Vaccine low and high responders have comparable frequencies of major circulating cell types

To test if differences in circulating immune cell frequencies and function could explain the differential induction of humoral and cellular immunity in response to vaccine response between low and high responders, we deployed mass cytometry on unstimulated, SARS-CoV-2 spike peptide-, and Cytostim-treated cells utilizing a custom panel of 40 antibodies against both surface and intracellular antigens (Supplementary Table 1). Projection of this multidimensional data onto uniform manifold approximation and projection (UMAP) embedded space revealed distinct clusters of cells corresponding to the major cell types within human peripheral blood mononuclear cells (PBMCs), identifiable by the expression of canonical markers (Supplementary Table 2): B cells, CD4^+^ T cells, CD8^+^ T cells, γδ T cells, NK cells, NK T cells, and myeloid cells (Fig. 1d). Comparison of frequencies of these major cell types between low and high responders did not reveal any significant differences, irrespective of vaccination or stimulation condition (Supplementary Fig. 1).

### Baseline differences in frequencies of B cell subsets are associated with vaccine responses

**Fig. 2:**
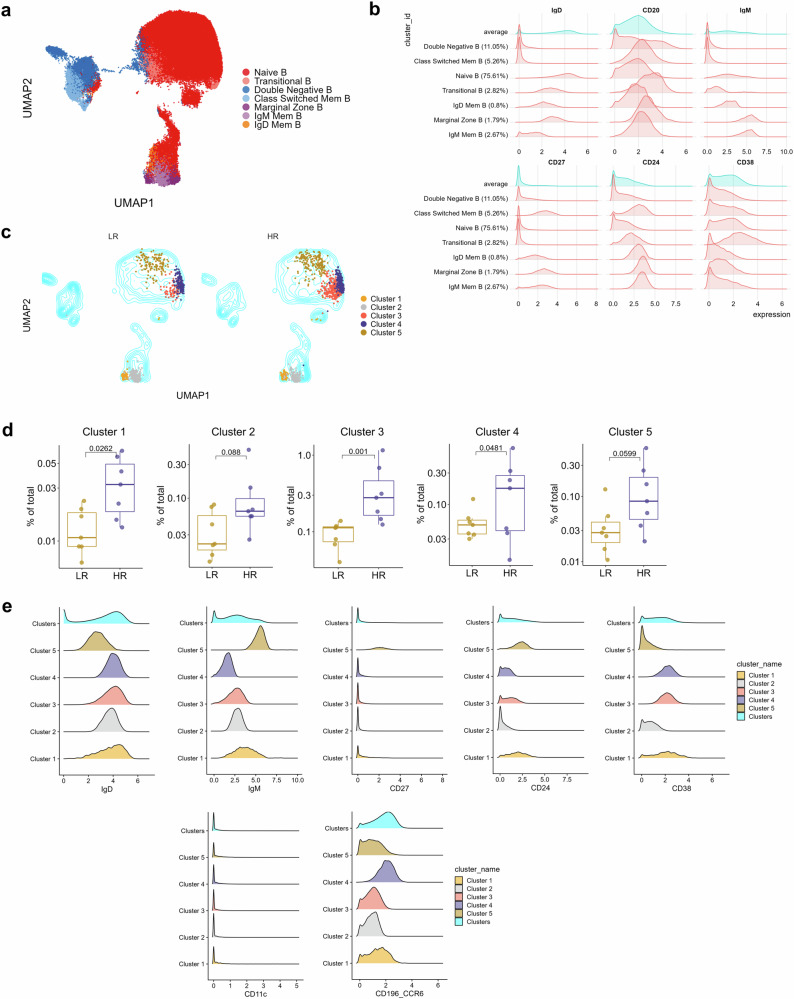
Low and high vaccine responders have differences in baseline B cell subpopulations frequencies. **a** UMAP plot of reclustered B cells depicting the major B cell populations based on expression of the markers denoted in (**b**). **b** Canonical B cell marker expression histogram to aid in the identification of the B cell subpopulations. **c** UMAP overlay of unsupervised clustering results for the B cells. The underlying contour plot is used to visualize all cells as in **a**. The overlaid dots represent the significantly different clusters between low and high responders. Only prevaccination samples are shown. **d** Boxplots of frequencies of significantly different B cell clusters between low (LR) and high (HR) responders in unstimulated pre-vaccination samples. Comparison of frequencies was done by modeling counts of clusters by negative binomial regression. Significance was assessed at *p* < 0.1 (FDR adjusted). The reported frequencies are calculated as % of total live PBMCs. **e** Expression histograms of canonical B cell markers for the five significant clusters observed in (**c**, **d**). For comparison, marker expressions for all the other clusters are also shown (‘Clusters’).

We tested whether preexisting differences in cell subset frequencies could discriminate between low and high responders to vaccination. We reclustered each of the major cell types based on the expression of all phenotypic and functional markers as visualized by UMAP (Supplementary Fig. 2). Initially, we wanted to investigate whether steady-state frequencies of B cell subsets can discriminate low and high responders. After reclustering the B cells, we identified seven distinct clusters based on the expression profiles of CD27, IgD, IgM, CD24, and CD38, and plotted them onto UMAP-embedded space to reveal phenotypically distinct subclusters within the B cell compartment (Fig. 2a). Visual inspection of expression histograms enabled the identification of canonical B cell subsets in circulation: CD27^-^IgD^+^CD24^+/-^CD38^-^ Naïve B cells, CD27^-^IgD^+^CD24^+^CD38^+^ Transitional B cells, CD27^-^IgD^-^ Double negative B cells, CD27^+^IgD^-^ class-switched memory B cells (SwM), CD27^+^IgD^+^IgM^+^ Marginal zone B cells, CD27^+^IgD^+^IgM^-^ non-class-switched memory B cells (IgD Mem B), and CD27^+^IgD^-^IgM^+^ non-class-switched memory B cells (IgM Mem B) (Fig. 2b, Supplementary Table 2). The frequencies of these subsets did not differ between low and high responders either before or after vaccination (Supplementary Fig. 3). However, deeper unsupervised clustering of B cells revealed smaller clusters whose frequencies differed between low and high responders before vaccination (Fig. 2c). Specifically, we observed that clusters bearing signatures of transitional (Cluster 1), naive (three phenotypically distinct subsets; CD38^-^, CCR6^-^ and CCR6^+^ subsets, Clusters 2, 3, and 4 respectively), and IgM^+^ non-class-switched memory B cells (Cluster 5) were significantly more abundant within the high responders in pre-vaccinated samples (Fig. 2d). The annotations were performed by inspecting canonical marker expressions for these significantly different clusters (Fig. 2e).

Taken together, our results indicate that higher baseline frequencies of subsets of mature and memory B cell populations are associated with high responders to SARS-CoV-2 vaccination.

### Helper T cell subsets respond differently to activation and antigen recall post-vaccination in low and high responders

**Fig. 3:**
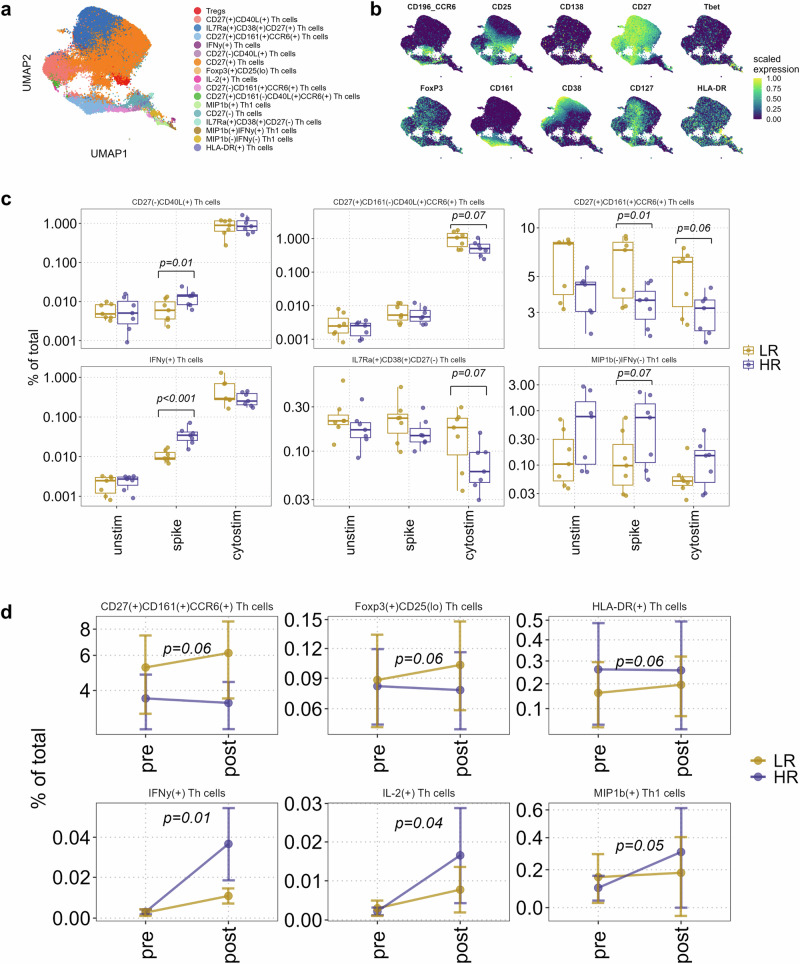
Vaccine low and high responders have differential CD4^+^ T cell response to activation. **a** UMAP plot of reclustered CD4^+^ T cells depicting the major populations based on expression of the markers denoted in B. **b** Canonical CD4^+^ T cell marker expression overlaid on the UMAP plot to aid in the identification of the subpopulations. **c** Boxplots of frequencies of significantly different CD4^+^ T cell clusters between low (LR) and high (HR) responders in post-vaccination samples. An FDR adjusted *p* < 0.1 was considered significant. The reported frequencies are calculated as % of total live PBMCs. **d** Line plots depicting the differential effect of vaccination on CD4^+^ T cell clusters between vaccine low (LR) and high (HR) responders. The plots show only significantly different clusters in spike-stimulated samples. The x-axis depicts pre- and post-vaccination status. Statistical significance was estimated by a negative binomial regression model. An FDR adjusted *p* < 0.1 was considered significant. Only significantly different clusters are shown for clarity. The reported frequencies are calculated as % of total live PBMCs.

Our next objective was to investigate differences in helper T cell responses between low and high responders. Reclustering of the CD4^+^ T cell compartment identified 17 distinct subsets including regulatory CD25^+^Foxp3^+^ T cells (Tregs), as well as smaller subsets of T_H_1 (CD4^+^Tbet^+^), and T_H_17 (CD4^+^CCR6^+^) cell types (Fig. 3a, Supplementary Fig. 4). Annotation of cell types was based on the expression of key surface antigens and transcription factors that are traditionally known to be associated with CD4^+^ T cell subtypes (Fig. 3b and Supplementary Table 2).

We observed a high degree of heterogeneity for spike peptide- and Cytostim-induced cytokine production between these cell types (Supplementary Fig. 4). In contrast to the B cell subsets, we found no significant differences in baseline CD4^+^ T cell subset frequencies between low and high responders (Supplementary Fig. 5a). However, after vaccination, unsupervised clustering revealed significantly different cluster abundances between low and high responders in both spike- and Cytostim-stimulated samples (Fig. 3c). We observed a significantly higher expansion of a subset of CD27^-^CD40L^+^ T_H_ cells in high responders with spike recall and a concurrent decrease in frequency of two subsets of CD27^+^CCR6^+^ T_H_ cells in high responders (Fig. 3c), one of which (CD27^+^CD161^-^) also expressed CD40L with cytostim (Supplementary Fig. 4). The more abundant of these two subsets was also positive for the NK cell receptor CD161 and showed a trend of decreased frequencies in the high responder group irrespective of stimulant (Fig. 3c). The high responders also had lower frequencies of an IL-7Rα^+^CD38^+^CD27^-^ T_H_ cell subset that was apparent for all stimulations but significantly different only for Cytostim, which seemed to induce a more pronounced difference in the abundance of IL-7Rα^+^CD38^+^CD27^-^ T_H_ cells between HR and LR. Concurrently, we observed a trend towards preferential increase of a Cytostim-induced MIP-1β^+^ T_H_ cell subset in HR compared to LR that did not reach our FDR cutoff (*p* = 0.11, Supplementary Fig. 5b). Additionally, we observed an expansion of IFN-γ^+^ T_H_ cells with spike stimulation, with high responders responding significantly more (Fig. 3c). Finally, a population of MIP-1β^-^IFN-γ^-^GzmB^+^ T_H_1 cells displayed a trend of higher frequencies in the high responders, but it reached statistical significance only with spike stimulated cells (Fig. 3c).

We then tested if vaccine-induced changes in the 17 CD4^+^T cluster cell frequencies were different between low and high responders. As shown in Fig. 3d, we observed significantly higher vaccine-induced expansions of IFN-γ^+^, IL-2^+^, and MIP-1β^+^ T_H_ cells in high responders after stimulation with spike peptides. Of the three, the IFN-γ^+^ and IL-2^+^ populations also expressed CD40L, while the IL-2^+^ population expressed low levels of Foxp3 and CD25 (Supplementary Fig. 4). On the other hand, a subset of the CD27^+^CD161^+^CCR6^+^ T_H_ cell type was preferentially expanded in the low responders upon vaccination. We also observed a vaccine-induced increase of a Foxp3^+^CD25^low^ regulatory T cell subset in the low responders.

A similar analysis to investigate differences in cytotoxic CD8^+^ T cell compartment did not reveal any cell subset that separated low and high responders, regardless of vaccination status or experimental activation (data not shown).

### Distinct subsets of γδ T cells may differentiate between low and high responders to SARS-CoV-2 vaccination

**Fig. 4:**
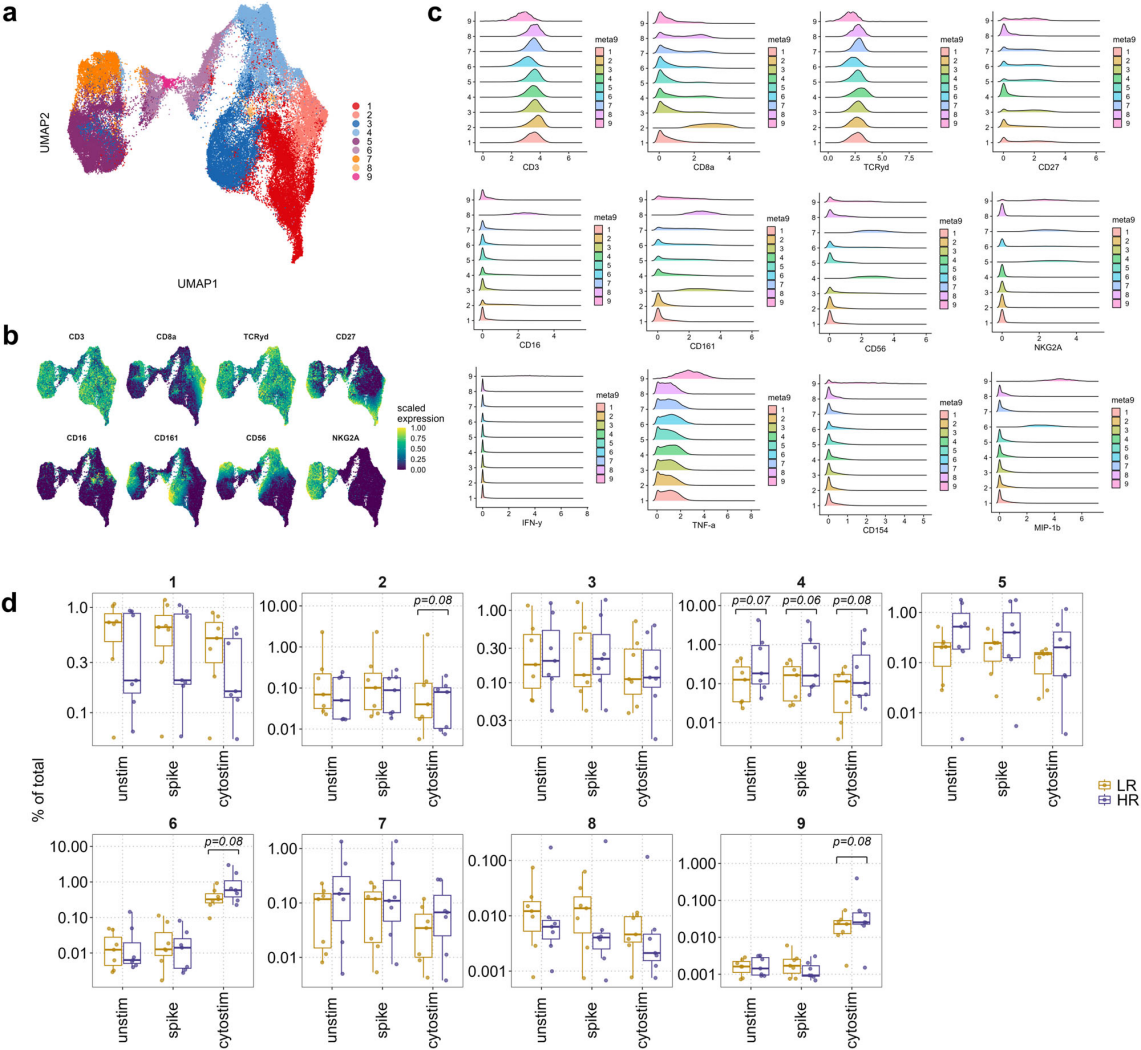
Differential abundances of γδ T cell subsets between vaccine low and high responders. **a** UMAP of reclustered γδ T cells colored according to the clusters identified by surface antigen expression. **b** Marker expression used for delineating the 9 metaclusters visualized in UMAP space. **c** Histograms of selected surface antigens and cytokines to establish the phenotype of the identified clusters. **d** Boxplots of frequencies of different γδ T cell subsets in low (LR) and high (HR) responders in post-vaccination samples being unstimulated, spike or Cytostim-stimulated in vitro. Comparison of frequencies was done by modeling counts of clusters by negative binomial regression. An FDR-adjusted *p* < 0.1 was considered statistically significant. The reported frequencies are calculated as % of total live PBMCs.

The role of γδ T cells in vaccine response is underexplored. Therefore, our next objective was to test if low and high responders to SARS-CoV-2 vaccination had different abundances of functionally different γδ T cell subsets. Reclustering of the total γδ T cell population revealed nine different subsets (Fig. 4a–c). In the pre-vaccination samples, we did not find any differences between low and high responders for these clusters (Supplementary Fig. 6). However, after vaccination we observed marked differences between the two response groups (Fig. 4d) for a CD56^+^ subset that was significantly more abundant in the high responders, regardless of the stimulation condition (cluster 4, Fig. 4c). We also found two small clusters within the γδ T cell compartment that expanded with Cytostim activation to be significantly higher in the high responders (clusters 6 and 9, Fig. 4d). These last two clusters were relatively lower for CD3 and TCRγδ expression (Fig. 4c), suggestive of their status as early γδ T cells. Moreover, cluster 9 also had high expression of the inhibitory receptor NKG2A and production of the cytokines IFN-γ, TNF-α, MIP-1β and CD40L (CD154) while cluster 6 was NKG2A^-^ but also exhibited MIP-1β expression (Fig. 4c). Among the other subsets, we identified a CD16^+^ cluster of γδ T cells which was lower in abundance in high responders across all stimulation conditions, although the difference did not reach statistical significance (cluster 8, Fig. 4d).

Taken together, our findings identified functionally distinct subsets of γδ T cells in the peripheral blood that differed between low and high responders to SARS-CoV-2 vaccination.

## Discussion

Since vaccines play a major role in the prevention of infectious diseases, inter-individual heterogeneity in vaccine response makes management of public vaccination policies challenging. This is even more of a concern in the elderly, whose weakened immune systems make them more vulnerable to infectious diseases^23^. Recent findings from our group identified epidemiological correlates of a SARS-CoV-2 vaccine-induced immune response in a cohort of Norwegian seniors over 65 years of age^16^. In the present study, we performed immune cellular mapping by mass cytometry to explore vaccine response among a subset of individuals from this cohort. Here we report differences between low and high vaccine responders for: (1) pre-vaccination frequencies of various B cell subsets, (2) response of CD4^+^ T cell subsets to antigen recall, and (3) frequencies of γδT cell subsets.

There have been many reports linking pre-vaccination frequencies of B cell subsets to immune response to a variety of vaccines in humans^24^. A common finding within these studies is a strong association of poor vaccine response with decreased frequencies of memory B cells. For example, increased age and consequent inadequate response to pneumococcal polysaccharide vaccines was shown to be associated with decreased frequencies of class-switched (CD20^+^CD27^+^IgD^-^) and non-class-switched memory B cells (CD20^+^CD27^+^IgM^+^IgD^+^), along with increased frequencies of naïve B cells (CD20^+^CD27^-^IgD^+^)^25^. Our results agree with the above observations, as we found frequencies of non-class-switched memory B cells to be lower in low-responding seniors. However, we did not find any difference in frequency of class-switched memory subsets. Moreover, we also found subsets of naïve B cells to be higher in the high responders. Another study reported that age-related inefficiency of a trivalent influenza vaccine response was associated with lower frequencies of transitional (IgD^+^CD27^+/-^CD38^+/-^), class-switched memory (SwM), and double-negative (DN) B cells^26^. We saw similar associations of lower frequencies of transitional and non-class-switched memory B cells with lower vaccine response. However, frequencies of double negative subsets were comparable between the vaccine response groups. While several findings were in agreement with age-related impairment to other types of vaccines, the observed discrepancies between our results and previous reports may be attributable to several factors, including that (i) in the above studies, comparison of the aged group was performed with a group of young adults as controls, whereas we measured differences between low and high vaccine responders within a cohort of elderly individuals, (ii) while previous studies compared differences in either polysaccharide or protein vaccines, we report responses to an mRNA vaccine. This latter distinction is important because different vaccines have inherently different mechanisms of immune activation. Polysaccharide vaccines, for example, do not involve a T cell response.

Baseline differences in T cell composition have been reported to influence responses to a respiratory syncytial virus (RSV) vaccine in the elderly^19^, where vaccine low responders had higher frequencies of activated (HLA-DR^+^) CD4^+^ and CD8^+^ T cells before vaccination. With the present experimental setup, we did not find any differences in subcluster frequencies between low and high vaccine responders within the T cell compartment in pre-vaccination samples. However, our results revealed differences in frequencies of several CD4^+^ T cell subsets post vaccination following restimulation with spike peptide. We found subsets of IFN-γ^+^ T_H_, CD40L^+^ T_H_ and a MIP1β^-^ IFNγ^-^ T_H_1 cells were significantly more expanded with antigen recall in the vaccine high responders as compared to the low responders, in accordance with a previous report^27^. Furthermore, we also noted vaccine high responders had lower abundances of CD161^+^ and CD161^-^ CCR6^+^ T_H_ cell subsets. This observation is significant considering a previous study that demonstrated increased CCR6^+^ T_H_17 cell subsets and circulating IL-17 in critically ill SARS-CoV-2 patients^28^. Additionally, we also observed that post-vaccination samples had higher CCR6^+^ T_H_ subsets in the low responders, whereas the high responders had higher frequencies of both IFN-γ^+^ and IL-2^+^ T_H_ subsets. There is evidence in literature for reciprocal regulation of T_H_1 and T_H_17 responses^29^. It has been shown in mouse models of autoimmune disorders that deficiency of either IFN-γ or IL-17 leads to T_H_17 or T_H_1 skewed cellular response, respectively^30^. The above observations raise the hypothesis that vaccine- or infection-induced increases in T_H_17 cell frequencies may hinder antiviral IFN-γ^+^ T_H_1 response. A recent study demonstrated that a better response to a conjugated polysaccharide vaccine against pneumococcal infections is associated with higher frequencies of T_H_1 cells and lower frequencies of T_H_17 cells^31^. Additionally, our observation that the low responders had higher vaccine-induced frequencies of a Foxp3^+^CD25^low^ regulatory T cell-like subset also substantiate the possibility of a dampened IFN-γ^+^ antiviral T_H_1 response. It would be interesting to probe if a T_H_1/T_H_17/Treg circuit has predictive value in determining vaccine response in the elderly. Moreover, we also observed that high responders have a higher induction of IL-2^+^ T_H_ cell subsets post-vaccination with a recall assay compared to their prevaccination frequencies than low responders. Further investigations are needed to demonstrate if high responders have better overall vaccine-induced effector T cell response.

γδ T cells play important roles in immune surveillance and tissue homeostasis^32^, but their role in vaccine response is not well studied. Evidence suggests that γδ T cells play a crucial role in responding to viral infections such as influenza, HIV, and cytomegalovirus (CMV)^33^. These cells can directly kill infected cells and expand in response to viral peptides. γδ T cells inhibit virus replication and promote immune responses. A recent study provided evidence of NK-like CD8^+^ γδ T cells as important mediators of immunity against persistent *Mycobacterium tuberculosis* infection^34^. Along similar lines, our data revealed a CD56^+^ population of γδ T cells were associated with high vaccine response. Two additional subsets of γδ T cells with significant cytokine-producing capabilities were found to be associated with better vaccine response. One of the clusters displayed high cytokine polyfunctionality by producing IFN-γ, TNF-α, CD40L upon activation with Cytostim. This is in agreement with a recent study that found an increase in Vδ2 cells producing TNF and IFN-γ upon MMR revaccination^35^. Additionally, higher production of TNFα by γδ T cells has been observed in the elderly^36^. Together, these results suggest a role of γδ T cells in mediating a high vaccine response in the elderly, which warrants further detailed investigations in larger cohorts and across a range of vaccination regimes.

In conclusion, the present study is to our knowledge the first attempt towards deep phenotypic and functional profiling of circulating immune cells associated with mRNA COVID-vaccine response heterogeneity in the elderly. Our results corroborate previous reports of pre-vaccination frequencies of mature and memory B cell subsets as predictive of subsequent vaccine responses. Moreover, we provide evidence of antigen recall-mediated expansion of cytokine-producing T cell subsets (both CD4 and γδT cells) as markers of better vaccine response. One notable strength of our study is the cohort which comprises a well-defined population of elderly individuals. However, our study is not without limitations. Owing to the small sample size and the exploratory nature of the work, we decided to choose a higher FDR threshold (0.1) than the usually practiced level of 0.05, as our aim in this study was hypothesis generation. This can be a potential limitation since some of the reported differences can turn out to be false positive events. Moreover, because of the small sample size, we chose not to adjust for any comorbidities that are often associated with elderly individuals. Therefore, further studies in larger cohorts are needed to inspect the effects of preexisting comorbidities on vaccine response heterogeneity. Our approach of calculating the abundance of immune cell subsets as a percentage of total PBMC bears the caveat that these percentages reduce the chances of finding compositional changes within immune cell lineages. However, our intent of reporting the frequencies as percentages of total PBMC was driven by our desire to capture a ‘systems-wide’ view of the cellular changes associated with vaccine response, which, in our opinion, is better suited for the type of discovery-based exploratory analysis performed in this work. We believe the broad conclusions should provide a basis for future studies for validation and possible mechanistic underpinnings of vaccine response in aged individuals.

## Methods

### Study cohort

In December 2020, the Norwegian Institute of Public Health established a population-based cohort directed towards people aged 65–80 yrs (The Senior Cohort). The aim was to measure the health consequences of the SARS-CoV-2 pandemic in this age group^16^. Initially, 1373 adults in the age group of 65–80 years and living in Oslo, Norway were randomly selected from the Norwegian Population Register and were invited to donate blood in the first round, of whom 488 provided consent. Blood samples were obtained from 412 participants, and PBMCs, serum, plasma, and DNA were isolated. Sampling was performed pre-vaccination (Dec 8, 2020, to May 4, 2021), and after the second vaccine dose (June 13 to Oct 27, 2021). All participants provided electronic informed consent. Participant data and national registry data were linked with the Norwegian unique personal identification number to obtain COVID-19 vaccination dates from the Norwegian Immunization Registry (SYSVAK); and dates of PCR-confirmed SARS-CoV-2 infections from the Norwegian Surveillance System for Communicable Diseases (MSIS). At the time of sampling, PCR testing and reporting to MSIS was mandatory. The post-dose 2 samples from the 14 participants included in this study were collected at least 14 days post-vaccination. None of the 14 participants had a record of SARS-CoV-2 infection at the time of sampling pre- and post-vaccination and were therefore considered uninfected. Subsequent analysis of anti-nucleocapsid (N) antibodies confirmed this observation, (i.e., anti-N antibodies were not detectable). Circulating antibodies against anti-RBD of SARS-CoV-2 spike protein were quantified as described previously^16^. Spike-specific T cell responses were quantified according to a previous report^16^. The Regional Committees for Medical and Health Research Ethics Southeast (229359) approved the study.

### Human peripheral blood mononuclear cells and cell stimulation

Thawed cryopreserved peripheral blood mononuclear cells (PBMC) (1–3 × 10^6^/well) were rested for 1 h at 37 °C in complete medium (RPMI 1640 Medium, 10% fetal bovine serum, 1 mM sodium pyruvate, 1% MEM non-essential amino acids, 12 μg/mL gensumycin (all ThermoFisher Scientific, Waltham, MA, USA), and 50 nM 1-thioglycerol (Sigma-Aldrich, Merck, Darmstadt, Germany), and then stimulated for 22 h with either SARS-CoV-2 spike peptides (Wuhan-Hu-1 strain; immunogenic (130-126-700) and complete (130-127-953), 0.3 nM/ml, Miltenyi Biotech, Germany), Cytostim (Miltenyi Biotech, Germany) or medium only (unstimulated). Brefeldin A (BD Golgi Plug, BD Biosciences, San Jose, CA, USA) was added after 2 h.

### Mass cytometry

Stimulated and non-stimulated samples were washed in PBS (Standard BioTools) prior to staining with Cell-ID™ Cisplatin-194Pt (Standard Biotools, 1:2000) for 10 min on ice. Cells were washed in Cell Staining Buffer (CSB (Standard BioTools)), Fc blocked (FcX, Biolegend) for 10 min at room temperature (RT) and each sample barcoded by staining with a unique mix of CD45 antibodies with three metals (Standard BioTools, Supplementary Table 1) for 30 min (RT). The individual barcoded samples were consolidated, counted (Muse® Guava®), and distributed into tubes of no more than 3 × 10^6^ cells/tube. The combined samples were stained with the surface antibody cocktail (Supplementary Table 1) in CSB for 30 min (RT) before being fixed using FIX I buffer (Standard BioTools) and permeabilized in methanol overnight (−20 °C). The next day the cells were washed in PBS and Perm-S buffer (both Standard BioTools) and stained with the intracellular antibody cocktail (Supplementary Table 1) in Perm-S for 30 min (RT). After staining the samples were washed in CSB and fixed using freshly made 1.6% formaldehyde in PBS (Thermo Scientific, Standard BioTools) for 10 min (20 °C). The samples were centrifuged and immediately resuspended in intercalation solution (0.5X Cell-ID Intercalator in Fix and Perm buffer, Standard BioTools) for 20 min (RT). The samples were washed twice in CSB, re-consolidated, and frozen in 10% DMSO in Fetal Calf Serum and stored at −80 °C. On the day of acquisition, the samples were thawed and washed twice in Maxpar® water (Standard BioTools) and filtered through a 35 μm cell strainer. The barcoded samples were acquired on a Helios mass cytometer (Standard BioTools).

### Data preprocessing

Raw mass cytometry data was bead normalized and concatenated using the CyTOF software (Standard Biotools). Initial preprocessing of normalized files involved filtering of debris through sequential gating of various Gaussian parameters, performed with the *CyTOFClean* package in R (https://github.com/JimboMahoney/cytofclean). Single cells were identified by gating on a bivariate plot of Ir-191 and Ir-193, followed by dead cell removal of Cisplatin-194 positive cells. Gating of single cells and exclusion of dead cells was done with a custom-written R script. Live single cells were then debarcoded using the *premessa* package in R (https://github.com/ParkerICI/premessa).

### Unsupervised clustering and annotation

Live single cells were initially clustered by FlowSOM with a 14 × 14 SOM grid and 30 meta-clusters, using all markers excluding the cytokines. The resulting clusters were visualized on UMAP-embedded space. For a coarse-grained analysis, the expression of lineage markers was visually inspected and the 30 meta-clusters were manually merged to obtain B cells (CD19^+^), CD4 T cells (CD3^+^CD4^+^), CD8 T cells (CD3^+^CD8^+^), γδ T cells (CD3^+^TCRγδ^+^), NK cells (CD56^+^), NK T cells (CD3^+^CD56^+^), and Myeloid cells (CD3^-^CD19^-^CD56^-^CD11b^+^). We also detected a very small fraction of cells that demonstrated inconclusive marker profiles (Undefined).

In the second step, further unsupervised clustering of each of the major cell types was performed by a 10 × 10 SOM grid and up to 80 meta-clusters starting with 2 metaclusters, using all markers including the cytokines. Thereafter, 20 metaclusters in each of B cells, CD4^+^ T cells, and γδ T cells were manually merged into 7, 17, and 9 annotated clusters, respectively, based on antigen expression visualized in marker histograms. Cell counts in each cluster per sample were then exported for further downstream analysis. FlowSOM clustering and UMAP visualization was done with the *CATALYST* R package. For annotating the clusters, we chose a combination of the natural separation of the cells by visual inspection of the UMAP embeddings and monitoring canonical marker expression in marker histograms. The scheme for annotation of the major clusters and their subtypes is listed in Supplementary Table 2.

Deeper unsupervised clustering was performed for the B cells, according to the following steps:

i. Significant differences between LR and HR were estimated at different metaclustering levels (*k* = 10, 20, 30, 40, 50, 60, 70, 80, and all 100 FlowSOM clusters).
ii. Then, unique clusters at each splitting step were selected by a marker similarity match. For example, if cluster no. 1 at *k* = 10 was phenotypically similar to cluster no. 5 at *k* = 20, then these two clusters were considered to be the same cluster. This way, we were able to identify clusters that are phenotypically unique at each value of k.
iii. The clusters significantly differing between the groups were FDR-adjusted.
iv. Steps i–iii were repeated three more times with different FlowSOM seeds.
v. All significant clusters across all FlowSOM runs were plotted on a UMAP plot, colored by the number of FlowSOM run. Only the clusters that appeared together in 3 out of 4 FlowSOM runs on the UMAP plot were finally reported to be significantly different (Supplementary Fig. 7).
vi. Significant clusters were annotated by examining marker expression histograms.

### Statistical analysis

Comparison of cluster frequencies between vaccine low and high responders within a given experimental condition was done by negative binomial regression of cell counts data using the total cells per sample as an offset, according to the following:

$${{cluster}}_{{count}} \sim {offset}\left(\log \left({{total}}_{{count}}\right)\right)+{vaccine\; response}$$

Significant differences between low and high responders were reported by adjusting for multiple testing by a false discovery rate (FDR) cutoff of <0.1.

For testing if vaccine-induced change in the frequency of a cell cluster was significantly different between low and high responders, we modeled the cell counts data of the cluster from post vaccination samples by regressing against vaccine response category, while offsetting for the frequency of the same cluster from the pre vaccination samples, according to the following:

$${pos}{t}_{{count}} \sim {offset}\left(\log \left({pos}{t}_{{total}}\right)\right)+{offset}\left(\log \left({pr}{e}_{{perc}}\right)\right)+{vaccine\; response}$$

To test for consistency across clustering owing to the high number of clusters tested for B cells, we performed FlowSOM clustering with four different seeds and tested for differences by negative binomial regression. Only clusters that were significantly different at least three out of the four FlowSOM runs were considered biologically different and thus reported. Negative binomial regression was done with the *Mass* package in R (v4.3.2).

## Supplementary information

Supplementary information

## Data Availability

The data analysed in this study come from a cohort of human volunteers and therefore contains information that may be perceived as sensitive. Due to protection of privacy and restrictions from the Norwegian Data Inspectorate and the Regional Committee for Medical and Health Research Ethics, the data are not publicly available. However, to facilitate reproduction of our analysis, encoded raw fcs files without attached metadata can be made available upon reasonable request to the corresponding author.

## Code availability

All computer codes for the analysis of data reported here are part of publicly available packages. The work does not generate new codes for analysis. R scripts used for data analysis can be made available upon reasonable request.
