# Supplementary material for: Mass cytometry reveals cellular correlates of immune response heterogeneity to SARS-CoV-2 vaccination in the elderly
Source: NPJ Vaccines. 2024 Nov 29;9:238. doi: 10.1038/s41541-024-01028-2 (PMC11607307; doi:10.1038/s41541-024-01028-2)
Supplement: Supplementary file 1 — Supplementary information [file 41541_2024_1028_MOESM1_ESM.pdf]

Supplementary Figure 1. Frequencies of major cell populations are not different between vaccine low and high responders

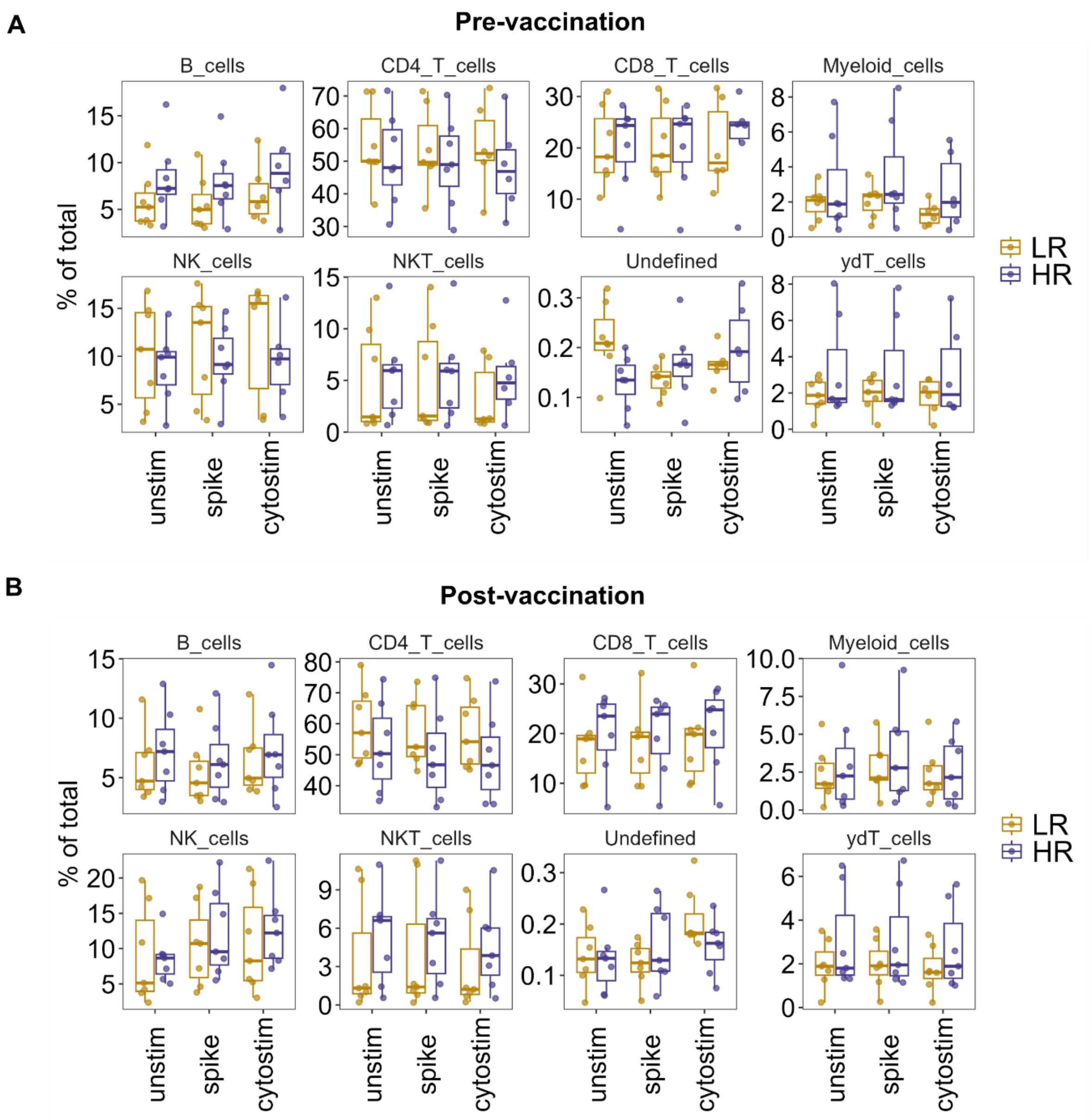

Boxplot of frequencies of major cell subsets in circulation in (A) pre-vaccination and (B) post-vaccination samples.

Supplementary Figure 2. Visualization of cell clusters by UMAP

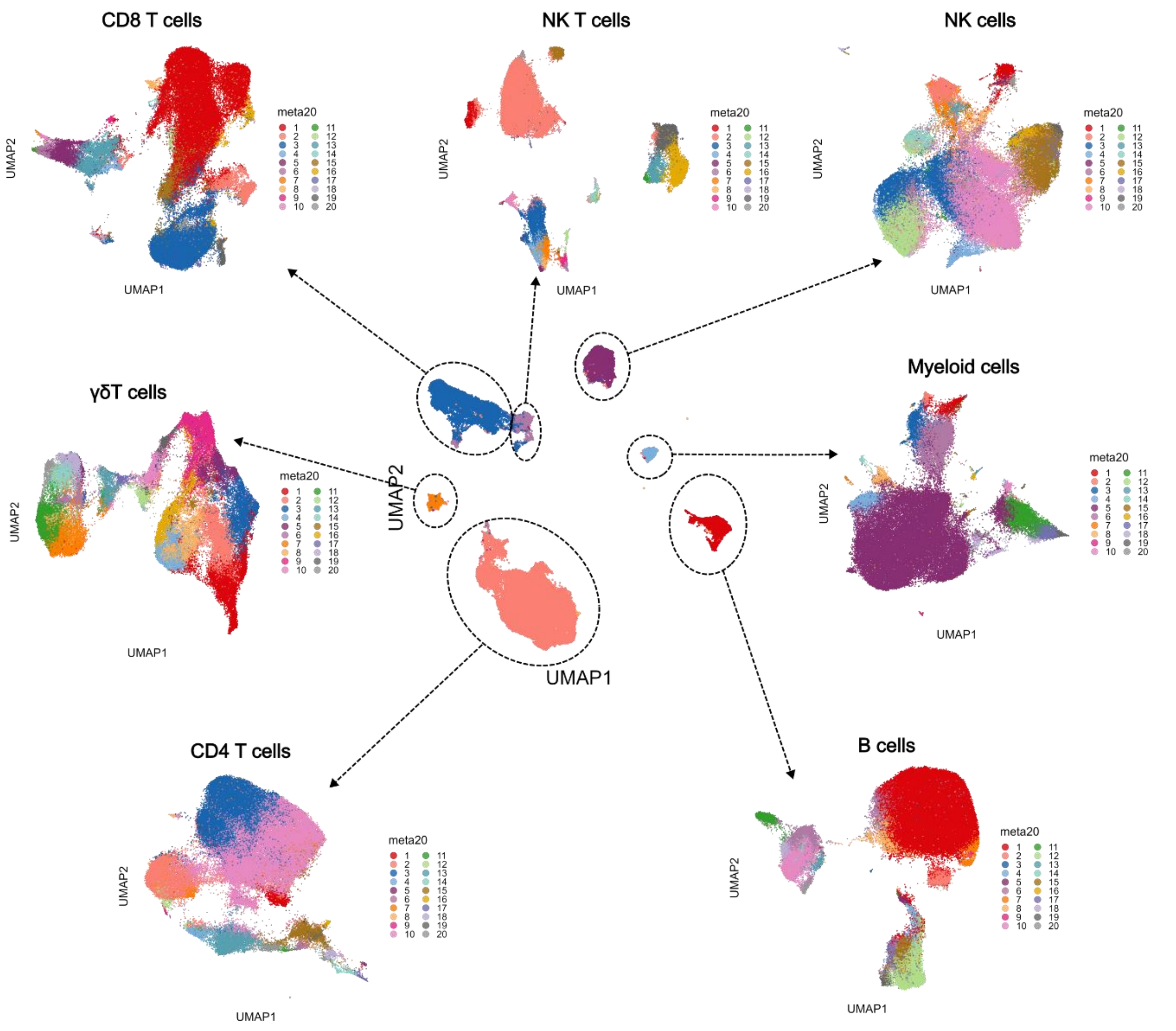

Annotated major cell populations (centre plot) were further reclustered and visualized by UMAP (plots on the periphery). For the sake of consistency of visualization, all the subsequent plots display 20 meta-clusters.

Supplementary Figure 3. Frequencies of major B cell subsets are not different between low and high responders to SARS-CoV-2 vaccine

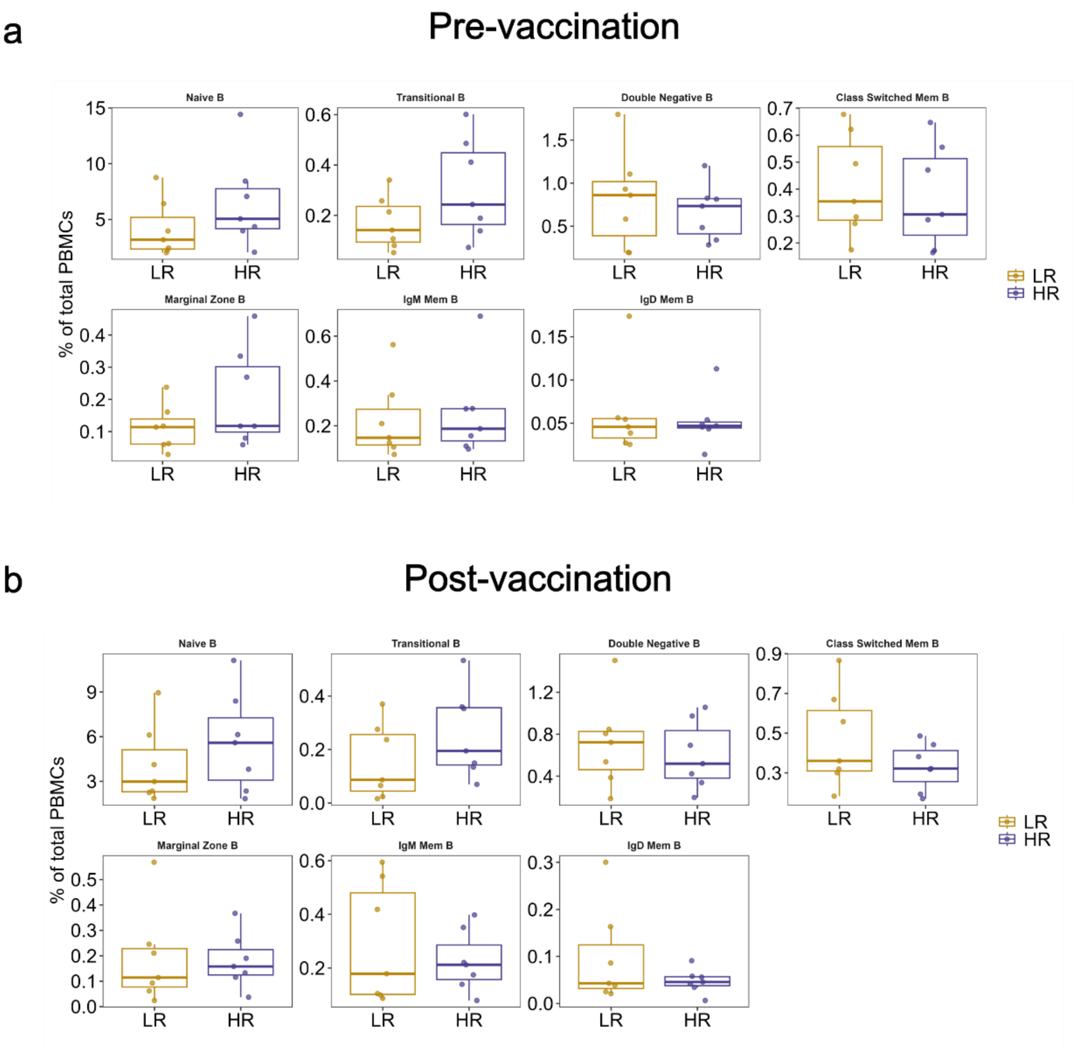

(a) Samples before vaccination. (b) Samples after 2nd dose of vaccination.

Supplementary Figure 4. CD4^{+} T cell subset annotation

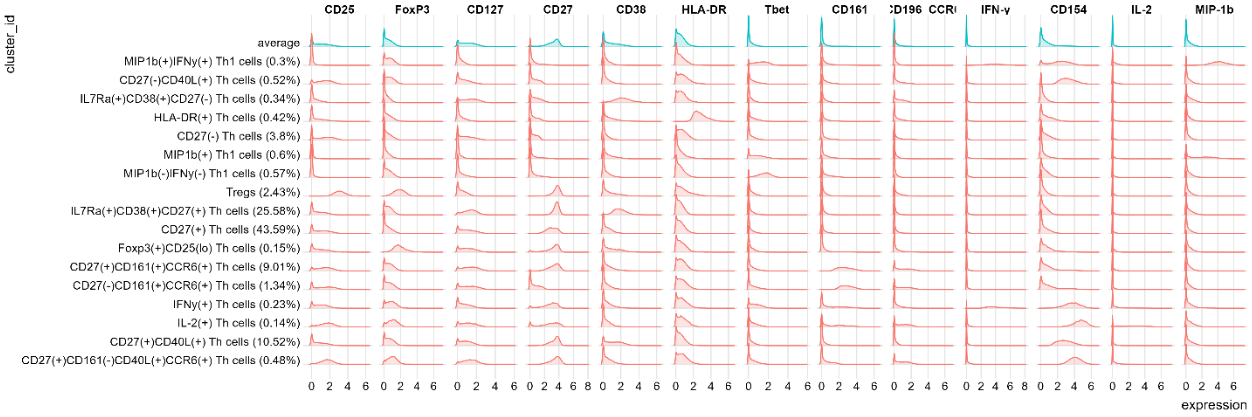

Histogram of phenotypic marker and cytokine expression of annotated clusters within the CD4^{+} T cell compartment in postvaccination samples across all stimulation conditions.

Supplementary Figure 5. Comparison of cluster frequencies and Cytostim-induced cytokine expression in CD4$^+$ T cells

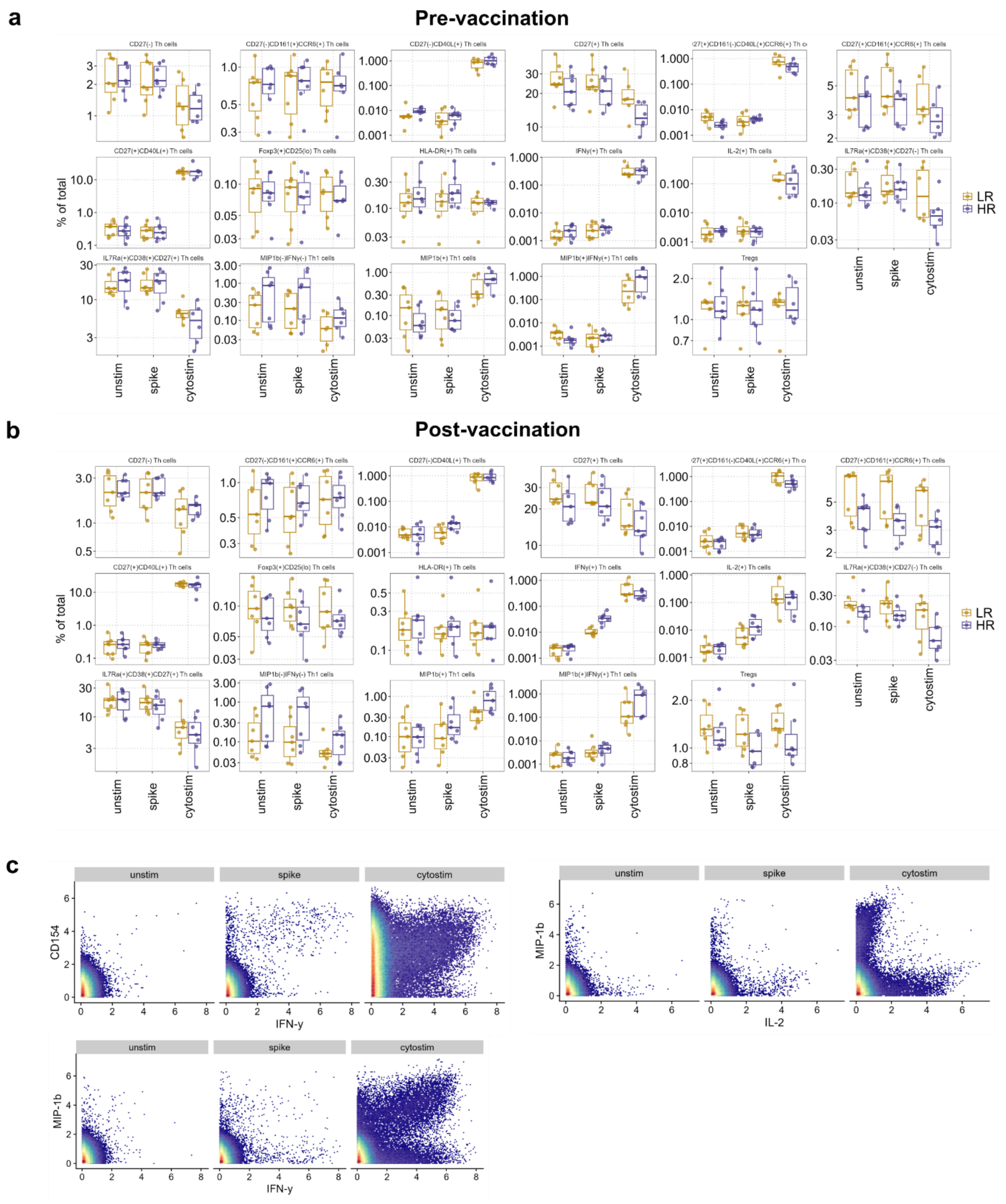

(a) Boxplot of frequencies of CD4$^+$ T cell subsets in vaccine low (LR, yellow) and high responders (HR, purple) in pre-vaccination samples for unstimulated, spike-stimulated, and cytostim-treated PBMCs.

(b) Boxplot of frequencies of $CD4^+$ T cell subsets in vaccine low (LR, yellow) and high responders (HR, purple) in post-vaccination samples for unstimulated, spike-stimulated, and cytostim-treated PBMCs.

(c) Representative dot plots of cytokine expression in total CD4 T cells in unstimulated, spike-stimulated, and cytostim-treated PBMCs.

Supplementary Figure 6. Frequencies of the γδ T cell subsets in pre-vaccination samples

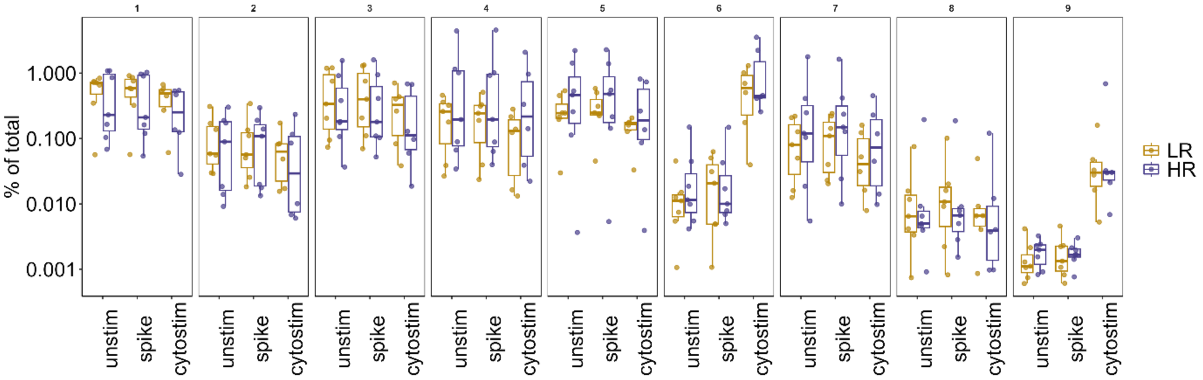

Boxplot of frequencies of the nine γδ T cell subsets in vaccine low (LR, yellow) and high responders (HR, purple) in pre-vaccination samples for unstimulated, spike-stimulated and cytostim-treated PBMCs.

Supplementary Figure 7. Comparison of significantly different B cell clusters across four different FlowSOM runs

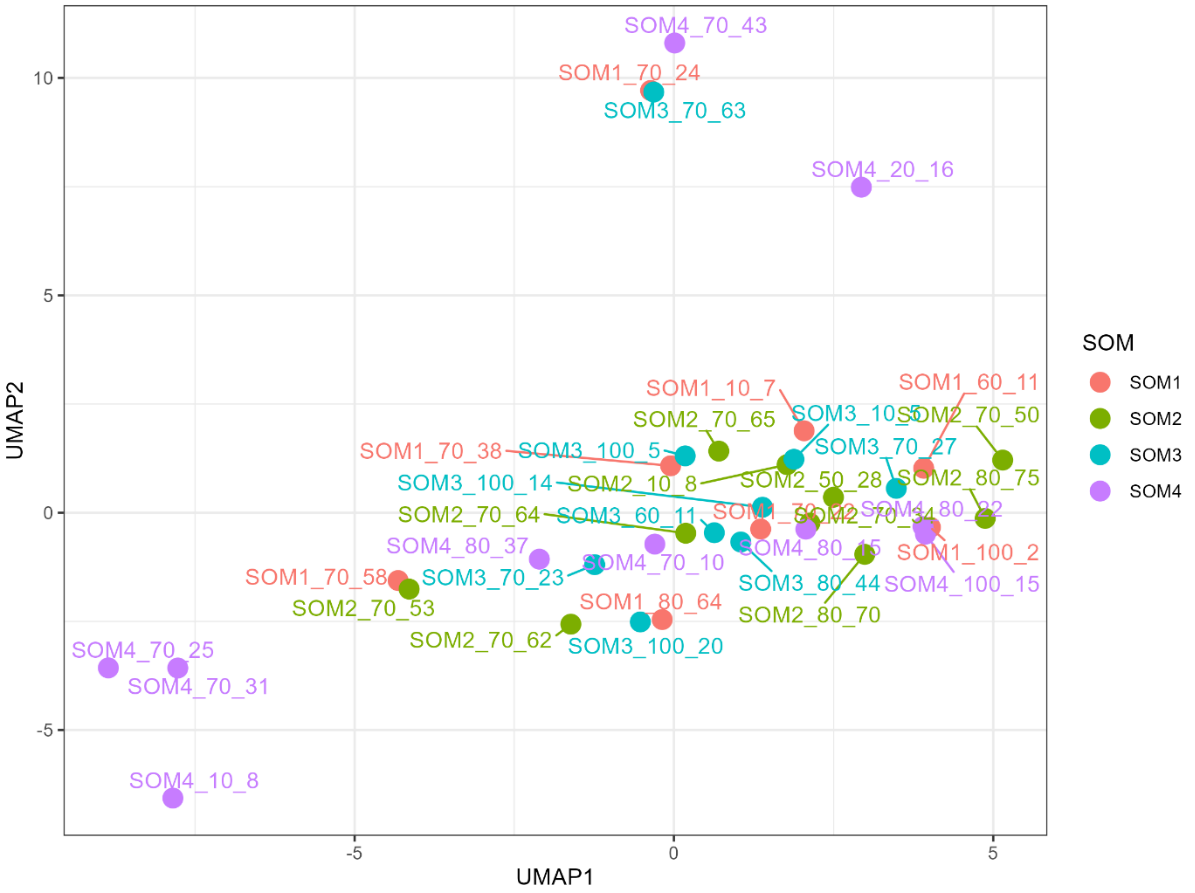

Each sphere is a B cell cluster that was found to be significantly different between LR and HR in prevaccination unstimulated samples and then projected onto UMAP space based on their marker expression profile. The colours represent the SOM run, each time with a different seed. Spheres close together on the UMAP space represent phenotypically same/similar clusters. Therefore, the three clusters on the bottom left are considered outliers since they appear only once out of the four SOM runs. On the other hand, the three clusters that appear at the top of the plot represent the same cluster that comes out to be significantly different in three out of the four SOM runs, and is therefore considered a true biologically relevant observation.

Supplementary Figure 8. Staining quality assessment of B cell markers

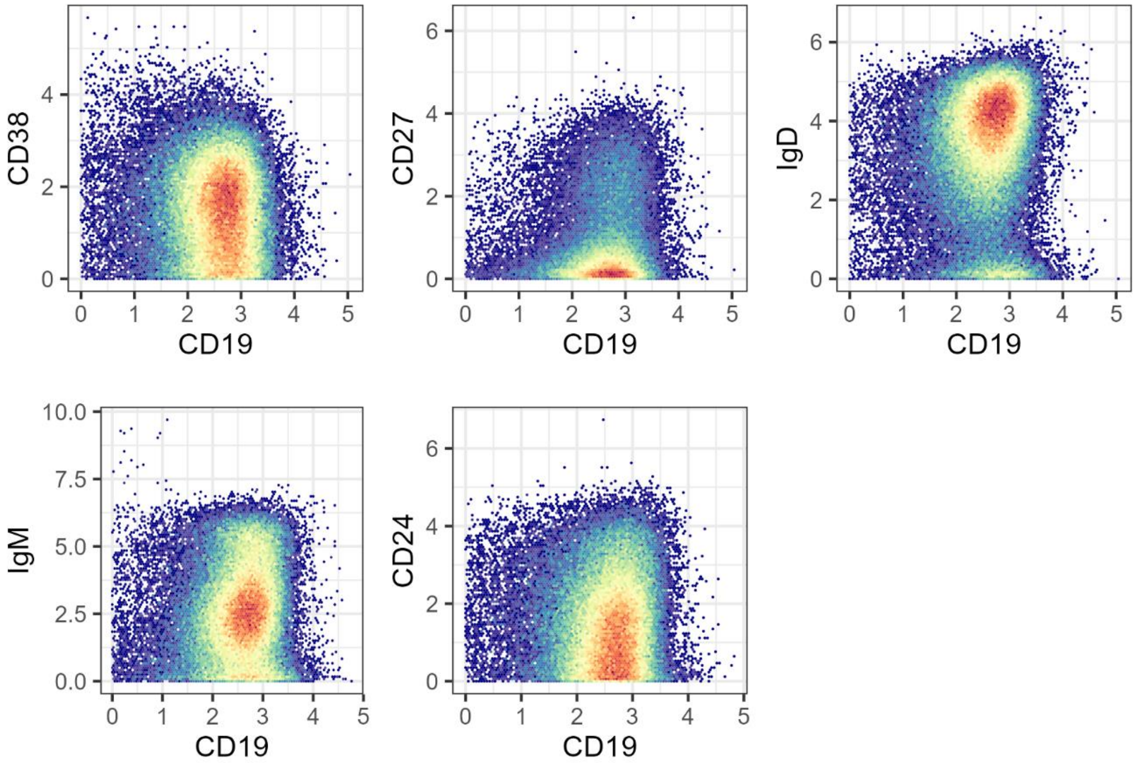

Bivariate scatterplots of markers used to annotate B cell subsets, plotted against CD19.

Supplementary Table 1. List of antibodies

Mass channel	Target	Clone	Catalogue No.	Supplier	Dilution
89Y	CD45 Barcode	HI30	3089003B	Standard Biotools	1/200
106Cd	CD45 Barcode	HI30	3106001B	Standard Biotools	1/200
110Cd	CD45 Barcode	HI30	3110001B	Standard Biotools	1/200
111Cd	CD19	HIB19	302247	Biolegend	1/400
113Cd	CD8a	RPA-T8	301053	Biolegend	1/800
114Cd	HLA-DR	L243	307651	Biolegend	1/200
116Cd	CD3	UCHT1	300443	Biolegend	1/100
141Pr	CD196 (CCR6)	11A9	3141014A	Standard Biotools	1/100
142Nd	IL-1b*	H1b-27	511605	Biolegend	1/200
143Nd	CD123	6H6	3143014B	Standard Biotools	1/200
144Nd	IgD	IA6-2	348235	Biolegend	1/800
145Nd	CD4	RPA-T4	3145001B	Standard Biotools	1/800
146Nd	TNFa*	Mab11	3146010B	Standard Biotools	1/100
147Sm	CD11c	Bu15	3147008B	Standard Biotools	1/400
148Nd	CD16	3G8	3148004B	Standard Biotools	1/400
149Sm	CD25 (IL-2R)	2A3	3149010B	Standard Biotools	1/100
150Nd	CD138	DL-101	3150012B	Standard Biotools	1/100
151Eu	CD107a (LAMP1)*	H4A3	3151002B	Standard Biotools	1/800
152Sm	TCRgd	11F2	3152008B	Standard Biotools	1/100
153Eu	IgM	MHM-88	314527	Biolegend	1/800
154Sm	IL-6*	MQ2-13A5	3154011B	Standard Biotools	1/100
155Gd	CD27	L128	3155001B	Standard Biotools	1/800
156Gd	CD86	IT2.2	3156008B	Standard Biotools	1/100
158Gd	IL-2*	MQ1-17H12	3158007B	Standard Biotools	1/100
159Tb	GM-CSF*	BVD2-21C11	3159008B	Standard Biotools	1/100
160Gd	MIP1beta*	D21-1351	3160013B	Standard Biotools	1/400
161Dy	Tbet*	4B10	3161014B	Standard Biotools	1/50
162Dy	Foxp3*	PCH101	3162011A	Standard Biotools	1/50
163Dy	CD56	NCAM16.2	3163007B	Standard Biotools	1/800
164Dy	CD161	HP-3G10	3164009B	Standard Biotools	1/100
165Ho	CD163	GHI/61	3165017B	Standard Biotools	1/50
166Er	IL-10*	JES3-9D7	3166008B	Standard Biotools	1/50
167Er	CD24	ML5	311127	Biolegend	1/200
168Er	IFNg*	B27	3168005B	Standard Biotools	1/200
169Tm	CD159a (NKG2A)	Z199	3169013B	Standard Biotools	1/100
170Er	CXCL10 (IP-10)*	R&D 33036	MAB266-100	R&D Systems	1/100
171Yb	CD20	2H7	3171012B	Standard Biotools	1/400
172Yb	CD38	HIT2	3172007B	Standard Biotools	1/100
173Yb	Granzyme B*	GB11	3173006B	Standard Biotools	1/400
174Yb	CD154 (CD40L)*	24-31	310835	Biolegend	1/100
175Lu	CD14	M5E2	3175015B	Standard Biotools	1/100
176Yb	CD127 (IL-7Ra)	A019D5	3176004B	Standard Biotools	1/100
191Ir	DNA	--	201192A	Standard Biotools	1/2000
193Ir	DNA	--		Standard Biotools	1/2000
194Pt	L/D	--	201194	Standard Biotools	1/2000
195Pt	CD45 Barcode	HI30	3195001B	Standard Biotools	1/200
196Pt	CD45 Barcode	HI30	3196001B	Standard Biotools	1/200
198Pt	CD45 Barcode	HI30	3198001B	Standard Biotools	1/200

Supplementary Table 2. Naming convention of cell types

**Cell type**	**Marker profile**
**B cells**	CD45+ CD3- CD19^{+}
Naïve B cells	CD27^{-}IgD^{+}CD24$^{+/-}$CD38^{-}
Resting naive B cells (resN)	CD27^{-}IgD^{+}CD24$^{+/-}$CD38^{-}CD11c^{-}
Transitional B cells	CD27^{-}IgD^{+}CD24^{+}CD38^{+}
Double negative B cells	CD27^{-}IgD^{-}
Class-switched memory B cells (SwM)	CD27^{+}IgD^{-}
Marginal zone B cells	CD27^{+}IgD^{+}IgM^{+}
Non-class-switched memory B cells (IgD Mem B)	CD27^{+}IgD^{+}IgM^{-}
Non-class-switched memory B cells (IgM Mem B)	CD27^{+}IgD^{-}IgM^{+}
**T cells**	CD45+ CD19- CD3^{+}
T_H	CD4^{+}CD25^{-}Foxp3^{-}
T_H1	CD4^{+}Tbet^{+}
Treg	CD4^{+}CD25^{+}Foxp3^{+}IL7Rα^{-}
**NK cells**	CD45+ CD3- CD19- CD56^{+}
**NKT cells**	CD45+ CD3- CD19- CD56^{+}CD3^{+}
**Myeloid cells**	CD3^{-}CD19^{-}CD56^{-}CD11b^{+}
